# Global trends in research on irritable bowel syndrome and the brain–gut axis: Bibliometrics and visualization analysis

**DOI:** 10.3389/fphar.2022.956204

**Published:** 2022-09-08

**Authors:** Peng-Ning Wu, Shuai Xiong, Peng Zhong, Wan-Qing Yang, Min Chen, Tai-Chun Tang

**Affiliations:** ^1^ School of Clinical Medicine, Chengdu University of TCM, Chengdu, China; ^2^ Department of colorectal diseases, Hospital of Chengdu University of Traditional Chinese Medicine, Chengdu, China

**Keywords:** irritable bowel syndrome, brain–gut axis, microbiome, bibliometrics, visualization analysis

## Abstract

Irritable bowel syndrome (IBS) is a gastrointestinal disorder with no structural damage, and its pathogenesis remains unclear. Studies have shown that the brain–gut axis is closely related to the occurrence of IBS. However, studies of IBS related to the brain–gut axis have not been systematically analyzed by bibliometrics and visual analysis. This study is based on 631 publications in the Web of Science Core Collection (WoSCC) to analyze hot spots and trends in this field. The collaborations between different authors, institutions, countries, and keywords were bibliometrically analyzed by CiteSpace software. Meanwhile, VOSviewer analyzed the references. The results show that since 2012, the number of publications has been growing rapidly. According to the collaborative network analysis, the United States, the National University of Ireland, Cork, and J.F. Cryan are the countries, institutions, and authors contributing the most, respectively. Through keywords and literature analysis, mechanisms and therapy associated with IBS and the brain–gut axis have still been a research focus in recent years. Furthermore, the physiological and pathological mechanisms of the brain–gut axis influencing IBS (related to gastrointestinal dysfunction, vagus nerve, visceral pain, intestinal flora, serotonin, tryptophan metabolism, stress, brain-derived neurotrophic factor (BDNF), and malonyldialdehyde) are the future research trends, especially the mechanisms related to intestinal flora. This is the first bibliometric and visualization analysis of IBS and brain–gut axis-related literature to explore research hotspots and trends.

## Introduction

IBS is a functional bowel disorder in which abdominal pain or discomfort is associated with defecation or a change in bowel habit and with features of disordered defecation (Longstreth et al., 2006). A systematic review and meta-analysis has demonstrated a global prevalence of IBS of 11% (Lovell & Ford, 2012), and its prevalence varies from ∼7.0 % in Southeast Asian and Middle Eastern studies to 11.8–14.0% in North American, North European, and Australasian studies and to 15.0–21.0% in South European, African, and South American studies (Black & Ford, 2020). Furthermore, IBS poses a significant disease burden, with 20% of adults estimated to develop IBS symptoms in any given year (Zyoud et al., 2021). The annual direct and indirect costs related to IBS are estimated to be up to €8 billion in Europe, ¥123 billion in China, and in excess of US $10 billion in the United States (Ford et al., 2020).

Since publication of the last British Society of Gastroenterology (BSG) guideline in 2007, substantial advances have been made in understanding its complex pathophysiology, resulting in its re-classification as a disorder of gut–brain interaction rather than a functional gastrointestinal disorder (Vasant et al., 2021). However, the mechanism of IBS is not fully clear. Its traditional mechanisms include the gut–brain axis, stress, visceral hypersensitivity, and altered motility (Martin et al., 2018). Since the microbiome has become an indispensable participant in gut–brain communication, researchers proposed the microbiota-gut–brain axis (MGBA) (Collins et al., 2012; Quigley, 2017; Cryan et al., 2019). Preclinical and clinical studies have shown bidirectional interactions within the brain–gut–microbiome axis (Mitrea et al., 2022). Gut microbes communicate with the central nervous system through at least three parallel and interacting channels involving nervous, endocrine, and immune signaling mechanisms (Martin et al., 2018; Mitrea et al., 2022). Evidence suggests a close relationship between the brain–gut axis and depression (Liang et al., 2018), autism (Saurman et al., 2020), Alzheimer’s disease (Kowalski & Mulak, 2019; Socała et al., 2021), Parkinson’s disease (Mulak & Bonaz, 2015; Caputi & Giron, 2018), epilepsy (Ding et al., 2021), hypertension (Yang et al., 2018), chronic kidney disease (Yang et al., 2018), inflammatory bowel disease (Gracie et al., 2019; Peppas et al., 2021), and multiple sclerosis (Parodi & Kerlero De Rosbo, 2021).

The metrological analysis of existing literature shows that the main fields of the MGBA include four research directions: “modeling the MGBA in animal systems,” “interplay between the gut microbiota and the immune system,” “irritable bowel syndrome related to gut microbiota,” and “neurodegenerative diseases related to gut microbiota” (Zyoud et al., 2019). Among them, the brain–gut axis has become the most prolific area for research within IBS and the microbiome globally (Zyoud et al., 2021). However, the association between IBS and the brain–gut axis has not been systematically studied through bibliometric and visualization analysis. Bibliometric analysis refers to use of mathematical and statistical methods to quantitatively analyze all the knowledge carriers of a certain discipline (Ma et al., 2020), playing an important role in reflecting characteristics and future trends. Based on WOSCC, CiteSpace and Vosviewer are used to perform bibliometrics and visualization analysis. This study aims to comprehensively and systematically review the current status of global IBS research related to the brain–gut axis from 2012 to 2021, making up for the lack of bibliometric analysis in this research area.

## Methods

### Data sources

**FIGURE 1:**
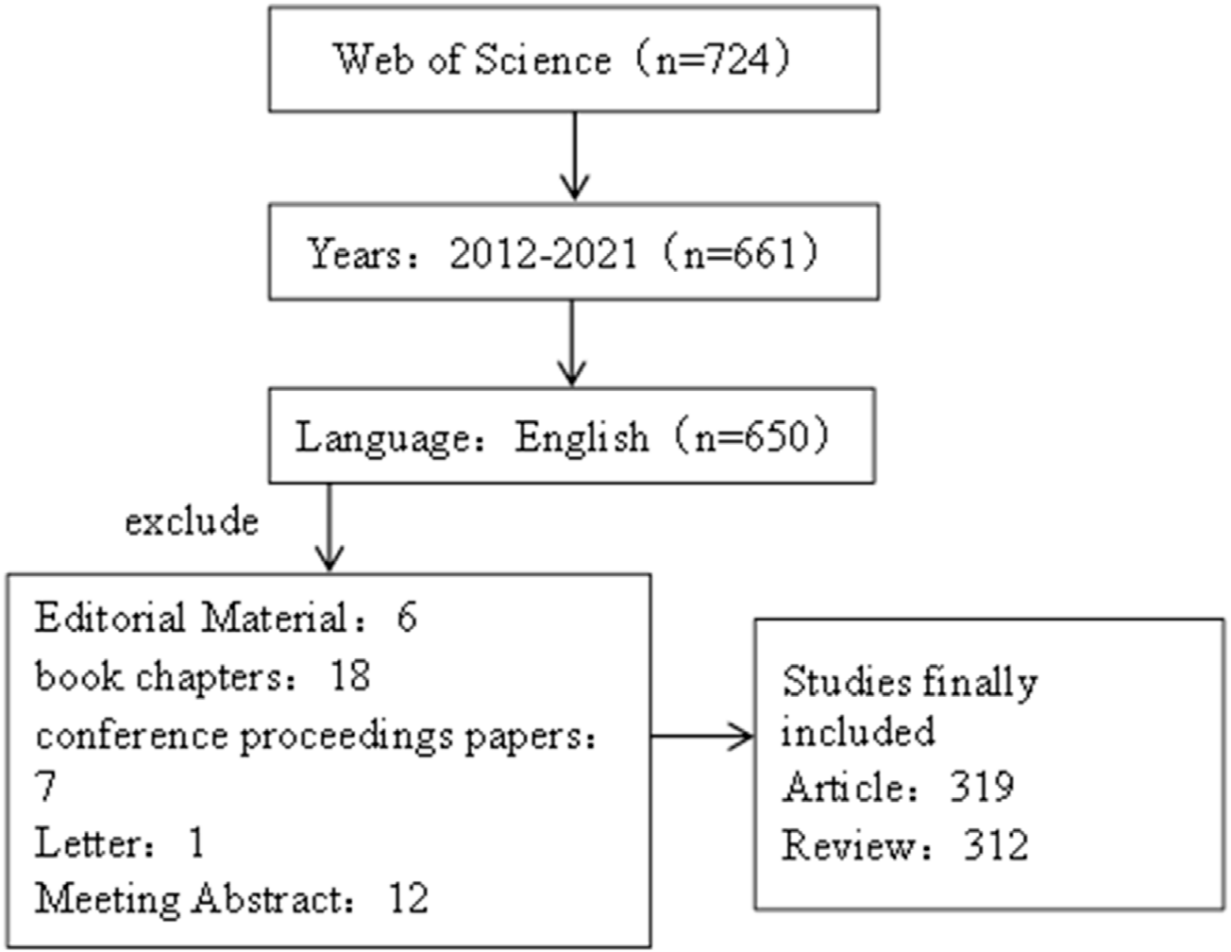
Flow diagram of the included articles.

The literature data for this study was obtained from the Web of Science Core Collection (WOSCC), and the retrieval language was set as “English”, and the period was set as “2012-01-01”–“2021-12-31”. At the same time, the retrieval literature type is set as “Article or Review”. The search criteria were subject and free words of irritable bowel syndrome and brain–gut axis. The specific search formula can be seen in the attachment (Search Strategy). Finally, the exported data include “full records and cited references,” and the records are exported as plain text files. The retrieval process was carried out independently by two researchers, and the retrieval date was 20 April 2022. Detailed retrieval results are shown in Figure 1.

### Data analysis

The software used for data analysis in this study is Microsoft Office Excel 2010, CiteSpace.5.8. R3, and Vosviewer1.6.17. Among them, Microsoft Office Excel 2010 is used for publication trend statistics, data sorting, and production of related tables. Also, CiteSpace.5.8 R3 is used to analyze the number of publications, mediating centrality of countries, institutions, and authors in the data and keyword frequency and mediating centrality, keyword outburst, and draw them into visual atlas, while Vosviewer1.6.17 is used to analyze highly cited and co-cited references. The specific parameter settings and result interpretation in CiteSpace have been described and published previously (Zou & Sun, 2021). The time slicing was set from the first day of 2012 to the last day of 2021. Node types include Author, Institution, Country, and Keyword.

## Results

### Publication trend analysis

**FIGURE 2:**
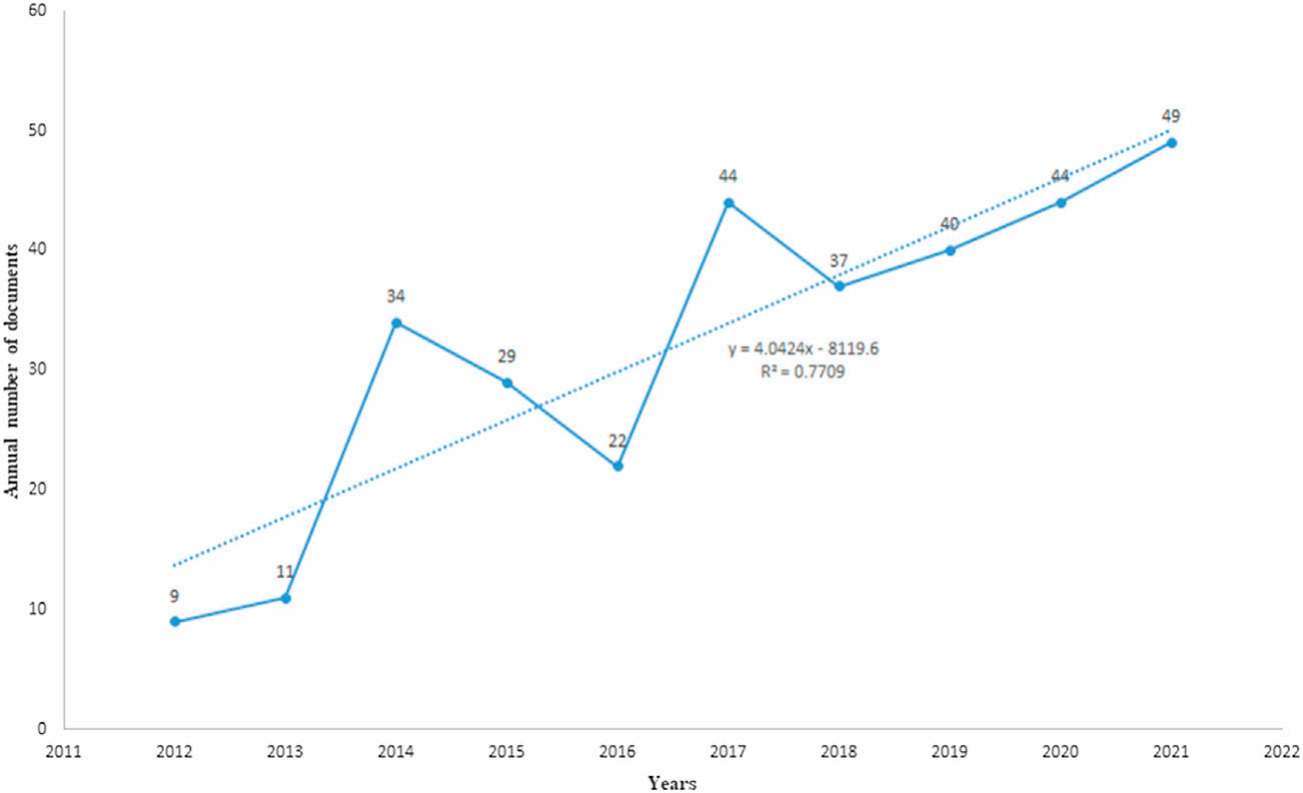
Published trend chart concerning IBS and the brain–gut axis. Analysis of countries/regions, institutions, and authors.

After WOSCC retrieval, 631 articles met the retrieval conditions, including 319 articles and 312 reviews. As shown in Figure 2, the number of articles published in 2012 was the lowest (nine articles), while the number of articles published in 2021 was the highest (49 papers). From 2012 to 2021, the number of articles about IBS and the brain–gut axis continued to increase steadily. It indicates that the study of IBS and the brain–gut axis has received more and more attention in the past decade, and more and more related studies have been conducted in this field.

**FIGURE 3:**
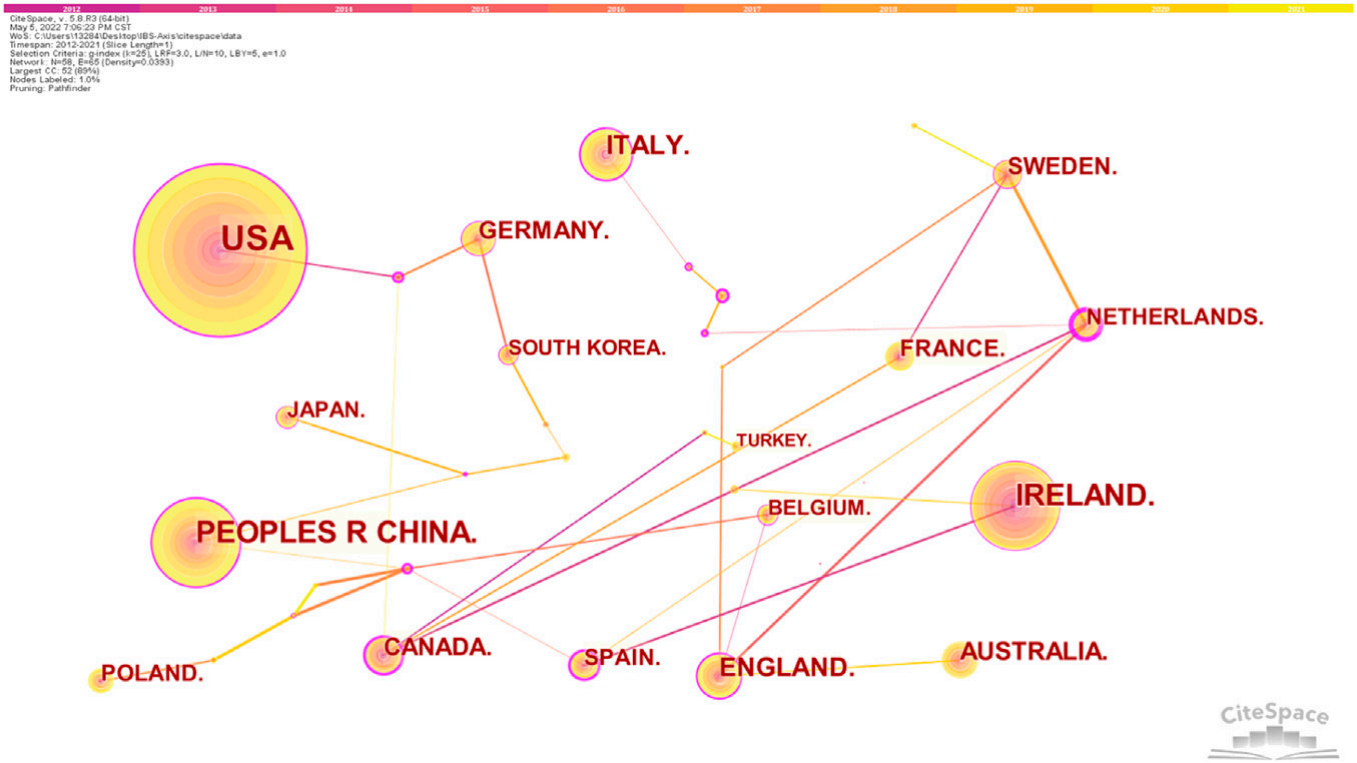
Country/region collaboration network of research on IBS and the brain–gut axis.

We observed the collaborative network of countries/regions, institutions, and authors for studies related to IBS and the brain–gut axis. The top 10 countries or regions by publication and centrality are shown in Table 1. At the same time, 58 nodes and 65 link lines are generated on the organizational network map (Figure 3). The larger the node is, the more articles are published. The outer circle of the point is represented in purple, with a centrality greater than 0.1. Also, the larger the centrality value, the more cooperation between nodes and other nodes. As shown in Figure 3, the United States contributed the most with the largest number of publications related to IBS and the brain–gut axis, followed by Ireland, People’s Republic of China, Italy, and England, indicating that these five countries are the core forces in the research field of IBS and the brain–gut axis. Also, the top five countries in centrality are the Netherlands, Spain, Brazil, Canada, and the Czech Republic. The Czech Republic only published two articles and the Philippines only published one article, but their centrality was 0.47 and 0.27, respectively. As shown in Table 1, Australia has a large number of documents, but the centrality is 0.00.

**TABLE 1:** Countries/regions, institutions, and authors ranked by publications and centrality.

Item	Ranking	Name	Publications	Name	Centrality
Country/region	1	United States	163	The Netherlands	0.95
	2	Ireland	84	Spain	0.59
	3	People’s Republic of China	83	Brazil	0.57
	4	Italy	48	Canada	0.52
	5	England	41	The Czech Republic	0.47
	6	Australia	35	Switzerland	0.44
	7	Canada	33	Israel	0.43
	8	Germany	31	Peoples R China	0.36
	9	France	28	Greece	0.34
	10	Sweden	26	Philippines	0.27
Institutions	1	Natl Univ Ireland Univ Coll Cork	86	Univ N Carolina	0.2
	2	Univ Calif Los Angeles	21	Natl Univ Ireland Univ Coll Cork	0.16
	3	McMaster Univ	19	Univ Gothenburg	0.15
	4	Univ N Carolina	15	Univ Groningen	0.14
	5	Maastricht Univ	7	Univ Calif Los Angeles	0.08
	6	Univ Duisburg Essen	7	CALTECH	0.08
	7	Mayo Clin	7	Univ Washington	0.07
	8	Baylor Coll Med	7	Acad Med Ctr	0.07
	9	Univ Newcastle	7	Ctr Integrat Psychiat	0.07
	10	Texas Childrens Hosp	6	Univ Newcastle	0.06
Authors	1	J F Cryan	68	J F Cryan	0.05
	2	Timothy G Dinan	51	Timothy G Dinan	0.05
	3	Gerard Clarke	24	Gerard Clarke	0.03
	4	Emeran A Mayer	10	Emeran A Mayer	0.04
	5	T G Dinan	10	T G Dinan	0.01

**FIGURE 4:**
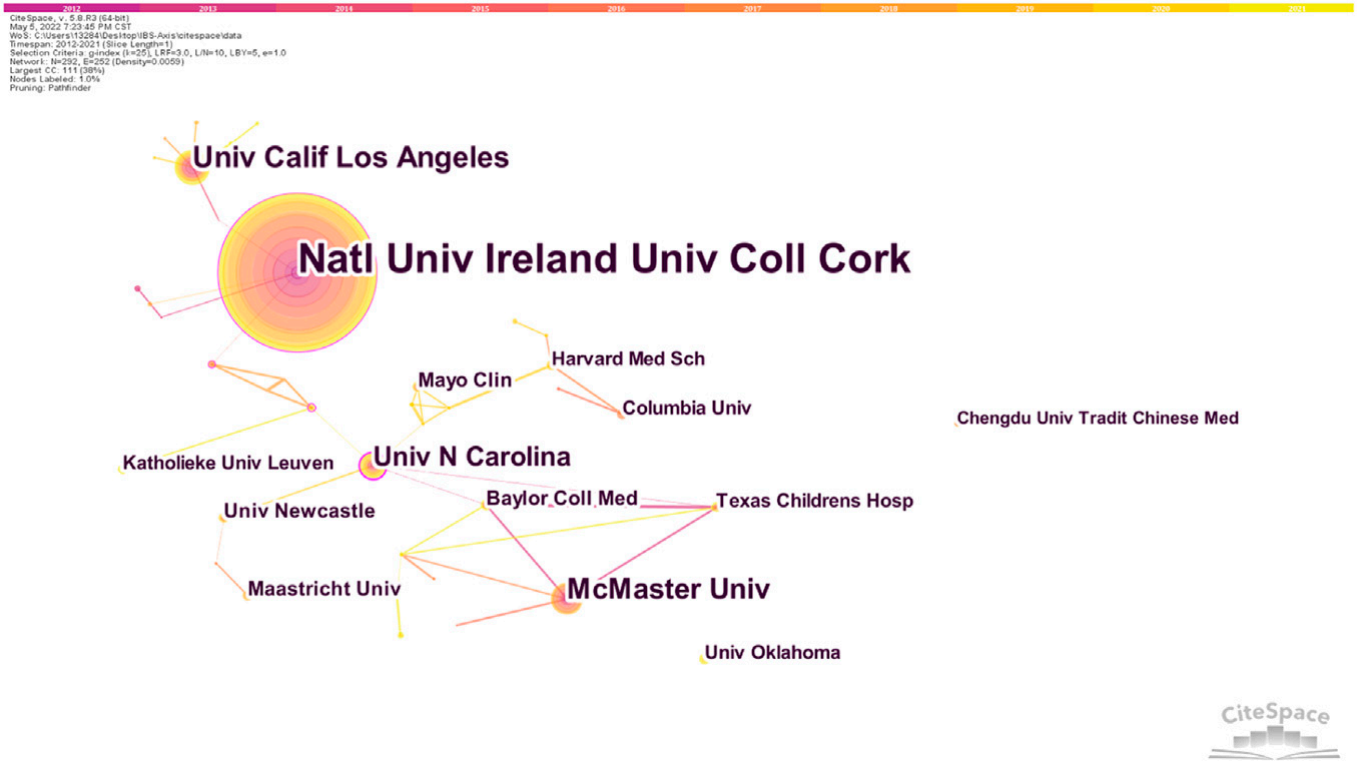
Institutions’ collaboration network of research on IBS and the brain–gut axis.

A cooperation network diagram of the published institutions is shown in Figure 4. 292 nodes and 267 link lines are generated on the organizational network map. The five institutions with the most publications are Natl Univ Ireland, Univ Coll Cork, Univ Calif Los Angeles, McMaster Univ, Univ N Carolina, and Univ N Carolina, while Natl Univ Ireland, Univ Coll Cork, Univ Gothenburg, Univ Groningen, and Univ Calif Los Angeles are the four institutions with the strongest centrality.

**FIGURE 5:**
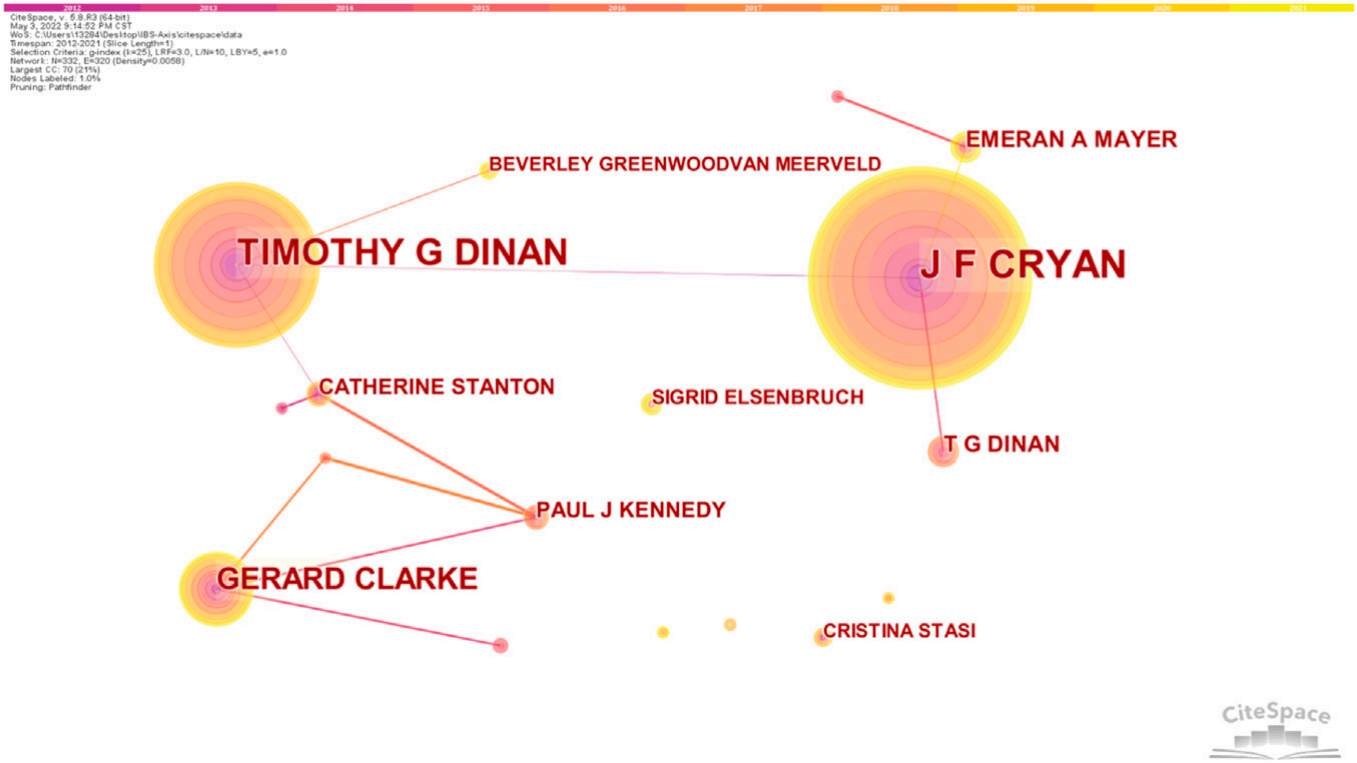
Author’s collaboration network of research on IBS and the brain–gut axis.

The author’s collaboration network of IBS and the brain–gut axis was observed. According to Figure 5, 332 nodes and 337 link lines are generated on the author’s network map. As shown in Table 1, there are three authors who have published more than 10 articles, with a centrality greater than 0.01. J F Cryan published the most articles (68 articles), followed by Timothy G Dinan (51 articles). The centrality of both of them reached 0.05. J F Cryan, Timothy G Dinan, Gerard Clarke, Emeran A Mayer, and T G Dinan are major contributors in the field of IBS and brain–gut axis research and have significant influence.

## Research topic analysis

### Research basis: analysis of highly co-cited references

**FIGURE 6:**
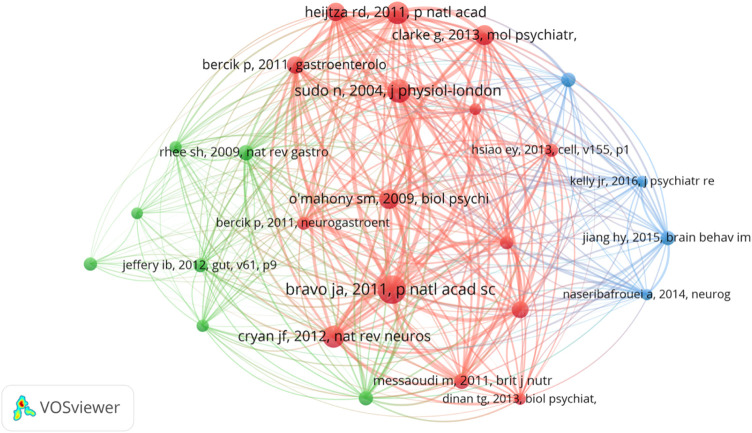
Visualization of a clustering map of highly cited references.

Co-cited references refer to the literature jointly cited by researchers. The purpose of co-citation analysis is to find the research basis of the common field between IBS and the brain–gut axis. We used VOSviewer to draw the co-citation reference graph, and the results showed that 37,025 co-cited references were cited. When the minimum citation time of cited references is set to 70, 26 literatures are left, and the final relational graph is shown in Figure 6. The network graph of highly co-cited references can be divided into three clusters, corresponding to three colors in the graph. The red cluster is mainly concerned with the interaction between intestinal flora and the central nervous system, including the influence of social stress on the immune function of intestinal microbiota (Bailey et al., 2011), the influence of intestinal microbiota on the central level and behavior of brain-derived neurotrophic factor in mice (Bercik et al., 2011), and the regulation of emotional behavior and central GABA receptor expression by *Lactobacillus* ingestion through the vagus nerve (Bravo et al., 2011). Literatures in the green cluster are more inclined to the relationship between intestinal flora and IBS and the treatment of IBS by probiotics regulating cytokines. Most of the literatures in the blue cluster are about the relationship between intestinal flora and depression. The top 10 literatures with the most co-cited times are shown in Table 2. It could be found that basic studies mainly focused on intestinal flora, emotional behavior, the adrenal system, brain and behavior, the hippocampal serotonergic system, early life stress, and central levels of brain-derived neurotropic factor. Among them, Javier A. Bravo published an article in 2011 that was co-cited the most, which found that lactic acid bacteria (such as *Lactobacillus* rhamnosus) can directly affect GABA (the main CNS inhibitory neurotransmitter) receptors in the central nervous system of normal healthy animals, thus participating in regulation of many physiological and psychological processes (Bravo et al., 2011). Changes in central GABA receptor expression are associated with pathogenesis of anxiety and depression, as well as with the pathogenesis of functional bowel disease. *Lactobacillus* rhamnosus (JB-1) reduces stress-induced corticosterone and behaviors associated with anxiety and depression by regulating GABA receptors. In addition, no neurochemical or behavioral effects were observed in mice with the vagus nerve severed, identifying the vagus nerve as the main regulatory component of communication between bacteria exposed to the gut and brain. Their study highlights the important role of bacteria in two-way communication on the brain–gut axis and suggests that certain organisms may prove to be effective adjunctive therapies targeting stress-related disorders such as anxiety and depression. This study lays a foundation for research on the influence of intestinal flora on mental illness and functional bowel disease.

**TABLE 2:** Highly co-cited references.

Item	Ranking	Title	Citation	Year
Co-cited references	1	Ingestion of *Lactobacillus* strain regulates emotional behavior and central GABA receptor expression in a mouse *via* the vagus nerve	182	2011
	2	Postnatal microbial colonization programs the hypothalamic–pituitary–adrenal system for stress response in mice	148	2004
	3	Normal gut microbiota modulates brain development and behavior	144	2011
	4	Mind-altering microorganisms: the impact of the gut microbiota on brain and behavior	141	2012
	5	The microbiome–gut–brain axis during early life regulates the hippocampal serotonergic system in a sex-dependent manner	127	2013
	6	Early life stress alters behavior, immunity, and microbiota in rats: implications for irritable bowel syndrome and psychiatric illnesses	127	2009
	7	Reduced anxiety-like behavior and central neurochemical change in germ-free mice	117	2011
	8	The intestinal microbiota affect central levels of brain-derived neurotropic factor and behavior in mice	111	2011
	9	Effects of the probiotic *Bifidobacterium infantis* in the maternal separation model of depression	106	2010
	10	Principles and clinical implications of the brain–gut–enteric microbiota axis	98	2013

### Analysis of co-occurring keywords, and burst term

**FIGURE 7:**
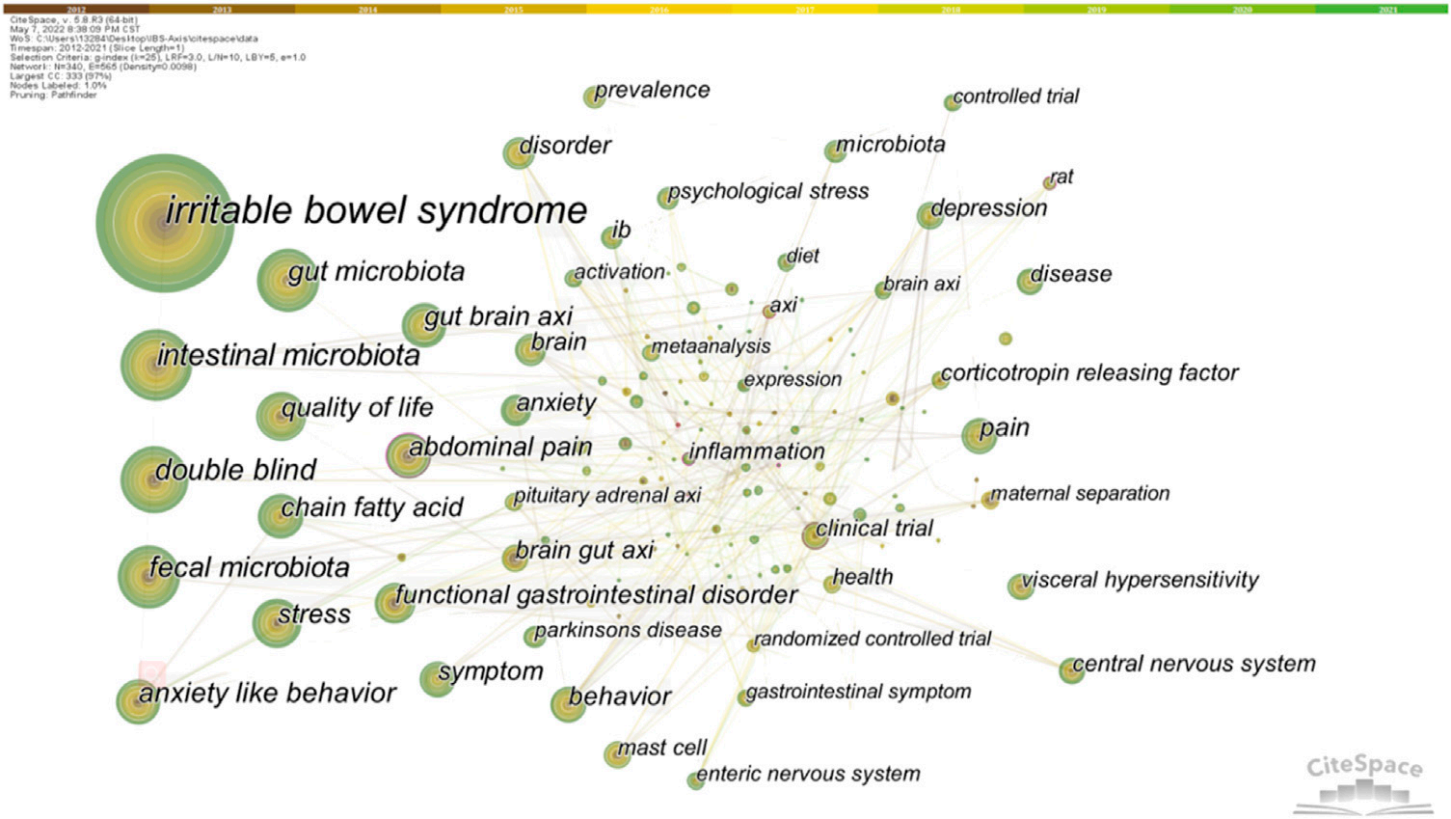
Co-occurring keywords map.

Keyword co-occurrence analysis can trace the research direction of a certain research field and quickly grasp the research topic. We made statistics on keywords related to the brain-gut axis of IBS from 2012 to 2021, and the top 20 keywords in statistical frequency and centrality are shown in Table 3. Obviously, “irritable bowel syndrome” was the most frequently used word, followed by microbiota, which includes the microbiota in the gut and feces. In addition, the frequency of “double blind,” “anxiety-like behavior,” “quality of life,” “chain fatty acid,” “stress,” and “gut brain axis” were all more than 50 times, revealing relevant research topics. Furthermore, the greater the centrality, the greater the bridge function of keywords in connecting two research fields. As shown in Table 3, “anxiety-like behavior” ranked first in centrality, followed by “Alzheimer’s disease,” “clinical trial,” “intestinal bacterial overgrowth,” and “inflammation”. It should be noted that these keywords combine IBS and the brain–gut axis with other studies. The keyword co-occurrence network diagram is shown in Figure 7.

**TABLE 3:** Top 20 keywords in terms of frequency and centrality.

Ranking	Keyword	Frequency	Keyword	Centrality
1	Irritable bowel syndrome	378	Anxiety-like behavior	0.32
2	Intestinal microbiota	92	Alzheimer’s disease	0.22
3	Double blind	78	Clinical trial	0.19
4	Anxiety-like behavior	73	Intestinal bacterial overgrowth	0.19
5	Fecal microbiota	68	Inflammation	0.17
6	Gut microbiota	59	gut	0.15
7	Quality of life	57	Abdominal pain	0.14
8	Short-chain fatty acid	57	Crohn’s disease	0.14
9	Stress	53	Rat	0.12
10	Gut–brain axis	52	Axis	0.11
11	Abdominal pain	48	Probiotics	0.11
12	Functional gastrointestinal disorder	45	Celiac disease	0.11
13	Symptom	42	Central nervous system	0.1
14	Behavior	41	Serotonin	0.1
15	Pain	40	Corticotropin releasing factor	0.09
16	Disorder	38	Colorectal distension	0.09
17	Brain	38	Rectal distension	0.09
18	Brain–gut axis	38	Neonatal maternal separation	0.09
19	Anxiety	37	Gamma aminobutyric acid	0.09
20	Depression	34	Stress	0.08

Keyword clustering can classify and summarize research topics. We used CiteSpace to cluster the keywords of IBS and brain–gut axis, resulting in 20 clusters. The first 14 clusters are shown in Table 4. In general, when the silhouette >0.7, the cluster is highly efficient and convincing (Zou & Sun, 2021). The results showed that the clustering average contour value S = 0.8429 (>0.7), indicating that the clustering effect is reasonable.

**TABLE 4:** Keyword cluster analysis (the silhouette value is over 0.7).

Cluster	Size	Sihouette	Mean year	Label (LLR)	Other keywords
#0	34	0.865	2014	Dysbiosis	Psychobiotic; intestinal microbiota; IBS; colonic dysfunction
#1	33	0.887	2015	Anxiety-like behavior	Gut; irritable bowel syndrome; visceral hypersensitivity; bile acid
#2	29	0.844	2015	IBS	Pain; visceral hypersensitivity; hypnosis; gut directed hypnotherapy
#3	27	0.851	2015	Leaky gut	Major depression; small intestinal bacterial overgrowth; migraine; prokinetics
#4	25	0.833	2015	Cognitive impairment	Disease; expression; ibs-d; s-ketamine
#5	22	0.838	2015	Depression	Substance p; amygdala; anxiety; toll-like receptors
#6	21	0.772	2015	Dyspepsia	Corticotropin-releasing factor; probiotics; sensory nerves; strain difference
#7	20	0.842	2017	Psychiatric illnesses	Hypothalamic–pituitary–adrenal axis; critical windows; brain plasticity; early life challenges
#8	19	0.811	2013	Anxiety	Mouse; autonomic nervous system
#9	19	0.902	2017	Tryptophan	Neurodegenerative disease; canine; microbial tryptophan metabolites; translational
#10	19	0.867	2016	Migraine disorders	Dementia; gut microbiota transplantation; receptor alpha; child
#11	18	0.806	2015	*Lactobacillus* reuteri	Brain–gut axis; quality of life; *Campylobacter jejunum*; occupational stress
#12	18	0.837	2019	Malonyldialdehyde	Schizoaffective disorders; gut microbiota dysbiosis; bidirectional communication; proinflammatory cytokines
#13	15	0.844	2015	Spinal dorsal horn	Neuron; receptor expression; colorectal distension; rat
#14	14	0.786	2014	GABA	Dendritic reorganization; suspended moxibustion; glucagon-like peptide-1 (glp-1); resveratrol

**FIGURE 8:**
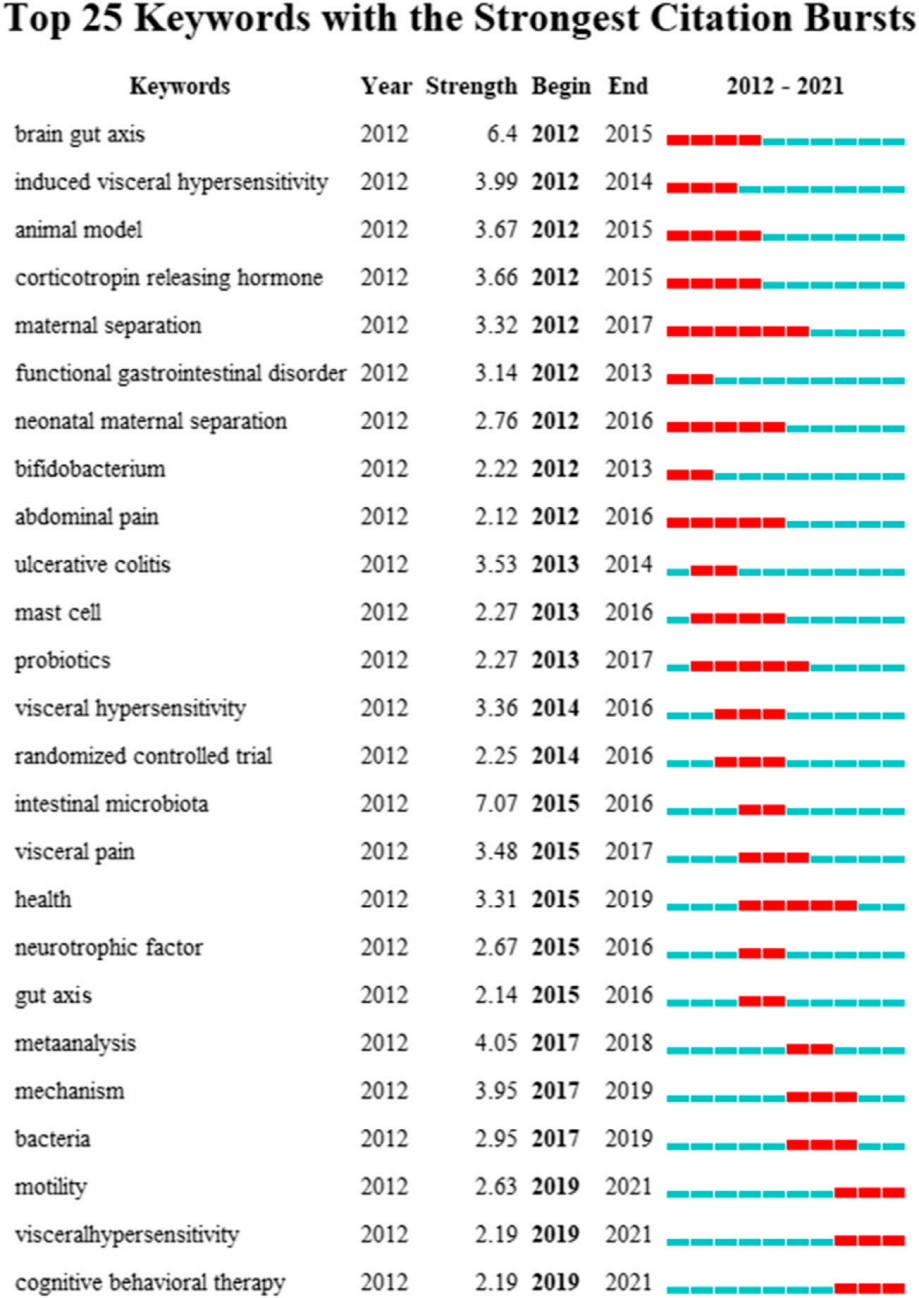
Top 25 keywords with the strongest citation bursts.

Similarly, keyword bursts can predict potential trends in a research field, implying a sudden increase in research content in a given period of time. Figure 8 shows 25 keywords with the strongest burst strength in this field. The blue line represents the time interval, and the red line represents the duration of the keyword burst. As can be seen, the key topics gradually developed from “induced visceral,” “animal model,” “corticotropin releasing hormone,” and “functional gastrointestinal disorder” to the current dimension of “meta-analysis” and “cognitive behavioral therapy” changes. This indicates that the research on IBS and the brain–gut axis in recent years is being carried out at the level of treatments.

### Analysis of highly cited references

We also revealed the top 10 highly cited references related to research on IBS and the brain–gut axis. As shown in Table 5, the most cited article was published in 2012 by J F Cryan et al. (Cryan & Dinan, 2012), which is a review of the impact of gut microbiota (GM) on the brain and behavior, concluding that GM plays a role in regulating anxiety, mood, cognition, and pain and that GM is associated with obesity, autism, and multiple sclerosis. In addition, this study summarizes the mechanism of the microbiome influencing gut–brain signaling, suggesting that the mechanism may include changes in microbial composition, immune activation, vagal nerve signaling, changes in tryptophan metabolism, production of neuroactive metabolites of specific microorganisms, and sugar of the bacterial cell wall. This review not only reflects the research trend but also lays the foundation for the study of the mechanism of IBS based on the brain–gut axis. In summary, the research frontier focuses on the microbiota in the brain–gut axis. These include the relationship between gut flora and brain and behavior, the relationship between the brain–gut axis and serotonin and tryptophan, and the relationship between gut flora and stress-related mental illness or depression, all of which reflect the latest research trends.

**TABLE 5:** Highly cited references.

Item	Ranking	Title	Citation	Year
Cited references	1	Mind-altering microorganisms: the impact of the gut microbiota on brain and behavior	2089	2012
	2	Microbiome–gut–brain axis during early life regulates the hippocampal serotonergic system in a sex-dependent manner	914	2013
	3	Interplay between the intestinal microbiota and the brain	850	2012
	4	Serotonin, tryptophan metabolism, and the brain–gut–microbiome axis	797	2015
	5	The Microbiota–Gut–Brain *Axis*	736	2019
	6	Gut/brain axis and the microbiota	667	2015
	7	Transferring the blues: depression-associated gut microbiota induces neurobehavioral changes in the rat	620	2016
	8	Psychobiotics: a novel class of psychotropic	530	2013
	9	Breaking down the barriers: the gut microbiome, intestinal permeability, and stress-related psychiatric disorders	484	2015
	10	Gut Microbes and the Brain: Paradigm Shift in Neuroscience	445	2014

## Discussion

### Publication trend analysis

From 2012 to 2021, the volume of literature on IBS and the brain–gut axis has gradually increased, indicating that this area is still a research hotspot. Clearly, research productivity in this area has increased due to increasing understanding of the role of GM. Among them, the United States is the most productive country in this field, and these findings are consistent with those of previous bibliometric studies (Zyoud et al., 2021). Massive financial support from the strength of the American economy and the motivation of researchers may be important reasons.

### Cooperative relationship

Bibliometric analysis can evaluate the cooperation of authors, institutions, and countries in specific research fields (Ma et al., 2020). The centrality value represents the cooperation intensity, and the higher the value, the stronger the cooperation. The Netherlands, Spain, Brazil, Canada, and the Czech Republic rank in the top five in centrality, indicating that they have close cooperation with other countries. However, the centrality of Australia is 0.00, indicating that Australia has little contact with other countries. Research into IBS in Australia may take a step toward promoting collaboration with other countries. In terms of collaboration in institutions, Natl Univ Ireland Univ Coll Cork, Univ Gothenburg, Univ Groningen, and Univ Calif Los Angeles cooperate most closely, with the strongest centrality. This indicates that the United States and Europe have an important influence in the study of IBS and the brain–gut axis. In terms of author collaboration, J F Cryan, Timothy G Dinan, Gerard Clarke, Emeran A Mayer, and T G Dinan work closely with other researchers. Collaboration among these authors may be associated with an interest in the field and substantial financial support for researchers.

### Research basis and hot spot

Bibliometric analysis can also help scholars grasp the development trends of specific research fields. According to the keyword burst analysis, it can be seen that people’s understanding of the influence of the brain–gut axis on IBS has gradually deepened, and the treatment of IBS based on the brain–gut axis is the current research focus. Also, the most commonly used keywords in the last decade can be preferred by researchers to access IBS and the brain–gut axis in this field. In addition, based on the references with high co-citation and high citation, we analyzed the research basis and Frontier related to IBS and the brain–gut axis. Studies have focused on the important role of intestinal flora in the brain–gut axis. Intestinal flora affects not only IBS but also metabolic diseases, central nervous system diseases, and psychological disorders through the brain–gut axis. For example, researchers have shown that ingestion of a *Lactobacillus* strain regulates emotional behavior and central GABA receptor expression in a mouse *via* the vagus nerve (Bravo et al., 2011). Also, colonization by GM impacts mammalian brain development and subsequent adult behavior (Diaz Heijtz et al., 2011). In short, studies of the effects of gut microbiota on the brain and behavior formed the basis of subsequent research.

### The relationship among IBS, the brain–gut axis, and gut microbiota

IBS is characterized by recurrent abdominal pain associated with abnormal stool form or frequency (Mearin et al., 2016). The traditional mechanisms of IBS include the brain–gut axis, stress, visceral hypersensitivity, and altered motility (Ford et al., 2020). The most studied part of the brain–gut axis is the GM.

GM is linked to other mechanisms through the brain–gut axis. Many other psychosocial, biological, and environmental factors are associated with IBS and might influence symptom severity. However, it is unclear if these are genuine risk factors because most studies are cross-sectional and do not have the temporal element needed to determine cause and effect.

The human GM is a complex, dynamic, and spatially heterogeneous ecosystem inhabited by a myriad of microorganisms interacting with each other and with the human host, including bacteria, fungi, archae, and viruses (Chen et al., 2021). The human GM plays a key role in a number of metabolic, nutritional, physiological, and immune processes through interactions with human hosts (Ottman et al., 2012). Once GM dysbiosis has adverse health effects on the human body, that will lead to a variety of chronic diseases (Chen et al., 2021).

### The relationship between gut microbiota and metabolic diseases

It is worth noting that through the analysis of literature related to IBS and the brain–gut axis, we found that the relationship between GM and metabolic diseases, such as obesity, is one of the research trends. GM and its metabolites can regulate satiety signaling and eating behavior by interacting with intestinal endocrine cells in the distal intestine (Lin et al., 2012). Comparisons of the distal GM of genetically obese mice and their lean littermates, as well as those of obese and lean human volunteers, have revealed that obesity is associated with changes in the relative abundance of the two dominant bacterial divisions, Bacteroidetes and Firmicutes. The obese microbiome has an increased capacity to harvest energy from the diet (Turnbaugh et al., 2006). Furthermore, GM after bariatric surgery (BS) appears to impact appetite regulation and satiety energy balance, thereby affecting weight control and metabolism (Martinou et al., 2022). Moreover, there is growing evidence that several natural herbs can effectively improve dyslipidemia by regulating gut flora (Jia et al., 2021), though their metabolic benefits and mechanisms are still not fully understood.

### The relationship between gut microbiota and central nervous system diseases

Intestinal flora is strongly correlated with central nervous system diseases and psychological disorders. Based on studies with experimental animals, significant progress has been made in the past decade in illuminating the role of bidirectional interactions between the GM, the gastrointestinal tract, and the nervous system (Margolis et al., 2021). The most studied interaction paths between the brain and the GM are represented by the endocrine pathway, consisting mainly of the HPA axis and enteric endocrine cells (EECs); the neural pathway, which includes the vagal nerve and the enteric nervous system; and the immune pathway, which is mediated via cytokines (Simon et al., 2021).

On the one hand, the imbalance of intestinal flora promotes neuroinflammation and plays an important role in the progression of Alzheimer’s disease (AD). A sodium oligomannate that has demonstrated solid and consistent cognition improvement in a phase 3 clinical trial in China suppresses gut dysbiosis and the associated phenylalanine/isoleucine accumulation, harnesses neuroinflammation, and reverses cognition impairment (Wang et al., 2019). On the other hand, antidepressants can effectively relieve symptoms in patients with IBS, and psychotherapy seems to be an effective treatment (Ford et al., 2019). These studies suggest that co-morbidity and co-treatment of gastrointestinal diseases, metabolic diseases, central nervous system diseases, and psychological diseases based on the brain–gut axis may be the direction of future research.

In summary, recent studies and clinical evidence have highlighted the importance of the gut microbiome in the pathophysiology of IBS, but the causality and translation of these findings in healthy individuals and patients with gastrointestinal or psychiatric disorders are limited. The specific benefits of using novel therapies such as prebiotics, probiotics, synbiotes, and fecal microbiota transfer (FMT) to regulate the GM in patients with IBS require further research (Simon et al., 2021). The mechanisms of how this broad microbiome affects human intestinal and central nervous system development (Obata & Pachnis, 2016), gastrointestinal motivity (Dimidi et al., 2017), mood (Simpson et al., 2021), cognition, and learning (Gareau, 2016) are still in their infancy, but it undeniably provides a potentially important site for future therapeutic interventions (Osadchiy et al., 2019). It provides directions for future studies of gastrointestinal diseases, metabolic diseases, and neurological diseases.

## Limitations

There are some limitations in our study that are similar to those of previous bibliometric studies (Zou & Sun, 2021; Zyoud et al., 2021). First of all, the WOSCC database was selected for our study, and only English articles were selected, which may result in some literature data missing; second, some authors or institutions have different name formats in the WOSCC database, which means their research counts may be scattered; third, this article does not ensure that every publication is completely relevant to the subject that meets the search criteria. Finally, it is not clear how the keywords and centrality were filtered to exclude terms that are not particularly relevant to IBS. For example, “double blind” and “clinical trial” are not particularly related to IBS. However, we believe that the results of our analysis are sufficient to reflect the overall state of the field.

## Conclusion

This study is based on bibliometrics and visualization analysis to assess and quantify a global overview of IBS and brain–gut axis-related research productivity from 631 publications on the Web of Science from 2012 to 2021. According to the analysis of the publications and references, the number of literature on IBS related to the brain–gut axis has continued to increase over the last decade. The United States, Ireland, China, Italy, and the United Kingdom are the countries that produce the most articles. Furthermore, it provides a comprehensive review of the progress of IBS and brain–gut axis research, identifies hot topics in the field, and points out potential directions for future research. According to the analysis of keywords and cited references, it can be found that the mechanism and treatment related to IBS and the brain–gut axis are still the focus of research in recent years. In addition, future research trends may include physiological and pathological mechanisms related to the brain–gut axis affecting IBS (related to gastrointestinal dysfunction, vagus nerve, visceral pain, intestinal flora, serotonin, tryptophan metabolism, stress, BDNF, and malonyldialdehyde), especially the mechanisms related to intestinal flora; moreover, this study found that the brain–gut axis is not only closely associated with IBS but also affects metabolic diseases, central nervous system diseases, and psychological diseases, such as obesity, Alzheimer’s disease, and depression, through intestinal flora. It suggests that future research can no longer be limited to studying the influence of the brain–gut axis on a single disease but to link diseases with shared mechanisms, explore deeper mechanisms, and find new directions for disease treatment. Furthermore, there are few literatures on different subtypes of IBS. In the future, studies on the relationship between the brain–gut axis and IBS can be divided into different subtypes. This timely review analyzes the results of IBS in relation to the brain–gut axis and may advance this field and lay the foundation for future research.

## Data Availability

The original contributions presented in the study are included in the article/Supplementary Material; further inquiries can be directed to the corresponding author.
